# Prognostic role of angiotensin-II receptor type 1 and endothelin-1 receptor type A agonistic autoantibodies in patients with acute myocardial infarction

**DOI:** 10.3389/fcvm.2025.1515693

**Published:** 2025-08-12

**Authors:** Francesco Tona, Giovanni Civieri, Marta Vadori, Giulia Masiero, Laura Iop, Martina Perazzolo Marra, Annagrazia Cecere, Marika Martini, Donatella Tansella, Giacomo Bernava, Benedetta Schiavon, Loira Leoni, Emanuele Cozzi, Sabino Iliceto

**Affiliations:** ^1^ Cardiology Division, Department of Cardiac, Thoracic, Vascular Sciences and Public Health, University of Padua, Padua, Italy; ^2^ Transplant Immunology Unit, Department of Cardiac, Thoracic, Vascular Sciences and Public Health, University of Padua, Padua, Italy; ^3^ Translational Biomedicine Group, Department of Cardiac, Thoracic, Vascular Sciences and Public Health, University of Padua, Padua, Italy; ^4^ School of Health, LUM-Libera Università Mediterranea “Giuseppe Degennaro”, Bari, Italy

**Keywords:** autoimmunity, no-reflow, coronary microcirculation, G protein coupled receptors, acute coronary syndrome, adverse prognosis, autoantibodies

## Abstract

**Background:**

Functional autoantibodies against angiotensin II type 1 (AT1R-AAs) and endothelin-1 type A (ETAR-AAs) receptors are associated with microvascular obstruction and myocardial remodeling after ST-elevation myocardial infarction (STEMI). However, their role in the long-term prognosis after STEMI has not been investigated.

**Methods:**

This is a prospective observational study enrolling STEMI patients undergoing early primary PCI. The incidence of major adverse cardiovascular events (MACE) was investigated during the follow-up. Autoantibody seropositivity was defined as a level >10 U/ml.

**Results:**

200 STEMI patients (89% male, median age 61 years) were enrolled. 110 (55%) were seronegative for both autoantibodies, 44 (22%) were seropositive for one autoantibody, and 46 (23%) were seropositive for both autoantibodies. Over a median follow-up of 1.2 years, the incidence of MACE was higher in patients with double (31%) and single (25%) seropositivity than in seronegative patients (13%, *p* = 0.02 among groups). Double seropositivity was independently associated with higher risk of MACE (HR 2.386, 95% CI 1.471–3.864, *p* < 0.001).

**Conclusion:**

AT1R-AAs and ETAR-AAs are associated with an increased risk of MACE after STEMI. Assessment of autoantibody levels paves the way for future therapies targeting specific molecular pathways associated with poor prognosis after an acute coronary event.

## 1 Introduction

ST–elevation myocardial infarction (STEMI) is a prevalent cardiac emergency that results in significant morbidity and mortality worldwide. The occurrence of this condition is similar in both Europe and the United States (1, 2). Although interventional treatment with primary percutaneous coronary intervention (PPCI) can markedly reduce short-term morbidity and mortality rates, the prognosis of STEMI remains relatively poor. In the last decades, no changes in the severity of myocardial damage and long-term outcomes have been observed (3).

In addition to their effects on blood pressure (4, 5), vasoconstrictor peptides, such as angiotensin II (AngII) and endothelin−1 (ET1), appear to be important contributors to the progression of heart failure following STEMI via non-hemodynamic pathways that lead to adverse myocardial remodeling. There is increasing evidence that these vasoconstrictor peptides contribute to the development of myocardial inflammation and fibrosis within both infarct and non-infarct regions, with the latter contributing to reduced myocardial elasticity and contractile dysfunction (6, 7).

AngII and ET1 exert detrimental effects by binding to the angiotensin II receptor type 1 (AT1R) and endothelin−1 receptor type A (ETAR), respectively. AT1R and ETAR are G protein-coupled receptors (GPCRs) expressed in a wide variety of cell types. Recently, functional autoantibodies against AT1R (AT1R-AAs) and ETAR (ETAR-AAs) have been identified (8). AT1R-AAs and ETAR-AAs appear to bind the same AT1R and ETAR receptors, permanently causing their hyperactivation (8). This leads to the development of an abiding pro-inflammatory environment with the formation of pro-inflammatory cytokines that participate in microvascular inflammation (9, 10).

While these autoantibodies have been documented in various clinical contexts, research on their impact on cardiovascular conditions is limited (11). However, AT1R-AAs and ETAR-AAs may play a significant role in numerous cardiac disorders, with their vasoconstrictive and inflammatory properties potentially contributing to poor outcomes following STEMI revascularization. Our recent findings indicate that ETAR-AA seropositivity is linked to no-reflow in patients with STEMI, and both ETAR-AAs and AT1R-AAs elevate the risk of adverse left ventricular remodeling after myocardial infarction (12, 13).

Since both no-reflow and adverse left ventricular remodeling negatively influence clinical outcomes after myocardial infarction (14, 15), we undertook the present study to analyze the prognostic role of AT1R-AAs and ETAR-AAs in patients with STEMI treated with PPCI.

## 2 Methods

This prospective observational study was conducted between January 2022 and June 2023. We enrolled 226 adult (>18 years) STEMI patients who had undergone reperfusion therapy by PPCI within 12 h of symptom onset at the Padua University Medical Center, expanding our previously described cohort and using the same inclusion and exclusion criteria (13). STEMI was defined as chest pain with (1) typical characteristics; (2) duration of more than 30 min; (3) an ST-segment elevation on initial ECG of >0.1 mV in two contiguous leads; (4) increased levels of cardiac troponin (cTn), with at least one measurement exceeding the 99th percentile upper reference limit. Reperfusion time was calculated as the duration between the initial onset of symptoms and crossing of the wire. Seven patients with a previous myocardial infarction, six with significant valvular heart disease, three with chronic atrial fibrillation, and three with inadequate image quality on transthoracic echocardiography (TTE) were excluded. The remaining 207 patients underwent serum testing for AT1R-AAs and ETAR-AAs. Patients were then followed up for a maximum of two years with clinically indicated in-person visits or telephone interviews. The physicians who cared for the patients were blinded to the autoantibody levels. Seven patients were lost to follow-up, and the final cohort comprised 200 patients.

### 2.1 PPCI procedure

All patients received guideline-directed medical therapy (250 mg aspirin and heparin (60 U/kg body weight) intravenously before PPCI; prasugrel (60 mg) or ticagrelor (180 mg) were chosen according to patient's age and weight. Aspirin was given indefinitely at a dose of 100 mg/day, while prasugrel (10 mg/day) or ticagrelor (90 mg twice daily) were continued according to the current guidelines (16). The use of glycoprotein IIb/IIIa inhibitors, beta-blockers, angiotensin-converting enzyme inhibitors, and statins was determined by the treating physician according to current guidelines (16). The TIMI study group classification (17) was used to grade the coronary flow in the infarct-related artery before and after revascularization of the culprit vessel. The myocardial blush grade (MBG) (0–3) before and after PPCI was also evaluated (18). Angiographic assessments were performed in the angiographic core laboratory by two blinded experienced cardiologists (F.T., G.M).

### 2.2 Echocardiographic analysis

LV end-diastolic volume index (LVEDVi) and end-systolic volume index (LVESVi) were measured using Simpson's biplane method. LV ejection fraction (LVEF) and wall motion score index (WMSI) were calculated as recommended by current guidelines (19). The mitral inflow peak early velocity (*E*)/mitral annular peak early velocity (*e*′) (*E*/*e*′ ratio) was also evaluated. Right ventricular function was measured as previously described (19). Additional details are provided in the Supplementary Methods.

### 2.3 Laboratory assays

Blood samples were drawn from a peripheral vein from all patients within 12 h of reperfusion. AT1R-AAs and ETAR-AAs were determined by enzyme-linked immunosorbent technique (ELISA) using a 96-well microtiter plate coated with AT1R and ETAR in their native configurations, according to the manufacturer's instructions (CellTrend, Luckenwalde, Germany). These kits are the most commonly used in studies on this topic (20–33). Seropositivity was defined using > 10.0 U/ml as the cutoff value for AT1R-AAs and ETAR-AAs, based on previous studies and following manufacturer recommendations (20, 22, 27, 29, 30, 33). Further details on the choice of the cutoff are reported in the Supplementary Methods. Patients were defined based on their serum autoantibody status as follows: (1) seronegative, when neither AT1R-AAs nor ETAR-AAs were above the cutoff value; (2) single seropositive, when either AT1R-AAs or ETAR-AAs (but not both) were positive; and (3) double seropositive, when both AT1R-AAs and ETAR-AAs were positive. Additional details in the Supplementary Methods.

### 2.4 Outcome definition

The study endpoint was the occurrence of major adverse cardiovascular events (MACE) during follow-up. MACE included cardiovascular mortality, nonfatal myocardial re-infarction, and hospitalization for heart failure. The time to the first event was considered for patients with more than one event during follow-up.

### 2.5 Follow-up and event adjudication

Patients were followed up for the occurrence of the study endpoint for up to 2 years (730 days) after enrollment. Structured follow-up was performed via telephone after inclusion. The endpoint was verified using medical records from the center, primary care clinicians, and other medical centers. A clinical endpoint committee consisting of two experienced cardiologists blinded to the autoantibody data reviewed and adjudicated all (possible) events.

### 2.6 Statistical analysis

The statistical packages R (version 4.3.1, R Foundation for Statistical Computing, Vienna, Austria) and IBM SPSS Statistics version 28 (Chicago, SPSS, Inc., Chicago, IL) were employed for statistical analysis. Continuous variables are presented as mean [standard deviation (SD)] if normally distributed or median [interquartile range (IQR)] if not normally distributed. Categorical variables are presented as numbers (percentages). Differences between two groups were analyzed using the Student *t*-test (for continuous variables with Gaussian distribution), the Mann–Whitney test (for continuous variables with non-parametric distributions), or Pearson *χ*^2^ test (for categorical variables). Differences between >2 groups were analyzed using one-way ANOVA for continuous variables with Gaussian distribution, the Kruskal–Wallis test for non-normally distributed continuous variables, and the Pearson *χ*^2^ test for categorical variables. Multiple comparisons of continuous variables were performed using the Bonferroni correction. After adjusting for related variables, multivariable logistic regression was used to analyze the association between continuous autoantibody serum levels and the risk of MACE. The resulting odds ratios are relative to a 1-unit increase in AT1R-AAs and ETAR-AAs levels. Survival curves were obtained using the R-package `survival’ (version 3.5.5) and compared using the log-rank test. The relationship between the continuous autoantibody levels and the risk of MACE was explored using Cox regression models by entering AT1R-AAs and ETAR-AAs as a restricted cubic spline, with three knots located at the 10th, 50th^,^ and 90th percentiles (for AT1R-AAs: 5.09, 7.67, and 15.3 U/ml; for ETAR-AAs: 4.14, 8.11, and 21.42 U/ml). Restricted cubic spline regression was performed using the R-package ‘rms’ (version 6.7.1). A univariate Cox proportional hazard regression model was used to estimate the hazard ratios (HR). A multivariable Cox proportional hazards regression analysis, using backward stepwise elimination (if *p* > 0.10), was performed to assess the risk factors independently associated with MACE. Possible confounders with a significant *p*-value in the univariate analysis were included in the multivariable regression analysis (*p* < 0.05) after checking for collinearity. Validity of assumptions for regression models were confirmed as appropriate. Model discrimination was assessed using *c-statistics*. All tests were two-sided, and the α level was set at 0.05. The authors had full access to and took full responsibility for the integrity of the data. All authors have read and agreed to the manuscript.

### 2.7 Ethical approval

This study was approved by the Institutional Ethics Committee (Comitato Etico per la Sperimentazione Clinica della Provincia di Padova; code number CESC 5478/AO) and was conducted in accordance with the Declaration of Helsinki and Italian laws. Informed consent was obtained from all patients included in this study.

## 3 Results

### 3.1 Clinical characteristics

The clinical characteristics, both of the overall population and divided according to seropositivity status, are reported in Table 1. A total of 110 (55%) patients were seronegative for both autoantibodies, 44 (22%) patients were seropositive for one of the two autoantibodies (AT1R-AAs or ETAR-AAs), and 46 (23%) patients were seropositive for both autoantibodies (AT1R-AAs and ETAR-AAs). Diabetes mellitus and known coronary artery disease had a different prevalence between the groups (*p* = 0.029 and *p* = 0.024, respectively) and were more frequent in patients with double seropositivity. The peak levels of troponin and BNP at admission were higher in seropositive patients (difference between groups, *p* = 0.046 and *p* = 0.001, respectively).

**Table 1:** Clinical and laboratory data for patients with STEMI (*n* = 200).

Baseline characteristics	Overall cohort (*n* = 200)	Seronegative patients (*n* = 110)	Single seropositive patients (*n* = 44)	Double seropositive patients (*n* = 46)	*p*
Demographic characteristics					
Age, years	62 ± 11	60 ± 10	58 ± 11	64 ± 13	0.138
Male sex, *n* (%)	166 (83)	91 (83)	35 (79)	40 (87)	0.641
Body surface area, m^2^	1.9 ± 0.2	1.8 ± 0.1	1.9 ± 0.4	1.8 ± 0.6	0.625
Body mass index, kg/m^2^	26 ± 4	27 ± 4	25 ± 4	27 ± 5	0.463
Medical history					
Obesity, *n* (%)	29 (14)	19 (17)	2 (4)	8 (17)	0.517
Hypertension, *n* (%)	97 (48)	51 (46)	22 (50)	24 (52)	0.783
Diabetes mellitus, *n* (%)	23 (11)	11 (10)	2 (4)	10 (22)	**0.029**
Hypercholesterolemia, *n* (%)	50 (25)	34 (31)	6 (14)	10 (22)	0.957
Smoker, *n* (%)	59 (29)	43 (39)	8 (18)	8 (17)	0.172
Ex-smoker, *n* (%)	79 (39)	56 (51)	10 (23)	13 (50)	0.314
Coronary artery disease, *n* (%)	15 (7)	8 (7)	0 (0)	7 (15)	**0.024**
Pacemaker, *n* (%)	5 (2)	2 (1.8)	1 (2.2)	2 (4.3)	0.858
Chronic obstructive pulmonary disease, *n* (%)	3 (1.5)	1 (0.9)	1 (2.2)	1 (2.1)	0.757
Peripheral vascular disease, *n* (%)	13 (6)	2 (1.8)	3 (7)	1 (2.1)	0.336
SBP at admission, mmHg	136 ± 24	136 ± 23	139 ± 29	138 ± 23	0.271
DBP at admission, mmHg	81 ± 15	81 ± 14	76 ± 19	79 ± 16	0.448
HR at admission, beats/min	81 ± 16	81 ± 15	85 ± 19	77 ± 17	0.121
O_2_ saturation at admission, %	97 ± 3	97 ± 3	98 ± 1	97 ± 2	0.323
Pain to balloon time, min	200 (125–393)	197 (124–404)	255 (147–385)	197 (122–306)	0.940
Door to balloon time, min	75 (47–125)	76 (48–120)	87 (41–110)	76 (46–115)	0.963
TIMI flow after PPCI <3	20 (10)	15 (14)	1 (2.3)	4 (9)	0.743
Blush grade after PPCI ≤2	16 (8)	13 (12)	0 (0)	3 (6)	0.400
Laboratory values at admission					
Peak of troponin, ng/L	64,061 (26,392–146,700)	53,665 (22,173–118,900)	89,474 (30,010–195,650)	73,911 (58,162–163,050)*	**0.046**
CRP, mg/L	5.35 (3–13.76)	5.30 (3.0–13.5)	10.95 (3.16–19.15)	11.62 (3.15–33.28)	0.173
BNP, pg/ml	112 (48–228)	107 (45–225)	117 (54–197)**	214 (69–389)**	**0.001**
eGFR, ml/min	63 (83–100)	93 (70–100)	84 (73–102)	91 (78–102)	0.476
D-dimer, ng/ml	150 (150–200)	150 (150–182)	150 (150–170)	164 (150–281)**	**0.002**
Hemoglobin, mmol/L	7.9 (6.8–9.7)	8.1 (6.7–9.8)	7.9 (6.7–9.1)	8.9 (7.0–8.8)	0.215
Creatinine, μmol/L	93 (80–115)	90 (80–110)	93 (81–111)	96 (83–114)	0.412
Urea, mmol/L	7.3 (6.3–11.4)	7.1 (6.2–10.8)	7.5 (6.8–12.1)	7.6 (7.0–11.6)	0.489
Medication at discharge					
ACE-inhibitor/ARB, *n* (%)	182 (91)	101 (92)	40 (91)	41 (89)	0.770
β-blocker, *n* (%)	134 (67)	78 (77)	18 (41)	38 (83)	0.076
Statin, *n* (%)	200 (100)	110 (100)	44 (100)	46 (100)	1.000
Diuretic, *n* (%)	12 (6)	6 (5)	2 (4.5)	4 (8)	0.305
Echocardiographic characteristics at discharge					
LVEDVi, ml/m^2^	57 ± 13	58 ± 16	58 ± 11	54 ± 9	0.730
LVESVi, ml/m^2^	31 ± 11	30 ± 12	30 ± 10	32 ± 8	0.648
LVEF, %	47 ± 10	49 ± 9	48 ± 8	45 ± 10	0.090
LVEF preserved ≥ 50%, *n* (%)	71 (35)	52 (47)	7 (16)	12 (26)	0.333
LV mass index, g/m^2^	97 ± 24	98 ± 25	87 ± 21	100 ± 22	0.521
Wall motion score index	1.69 ± 0.36	1.65 ± 0.33	1.69 ± 0.34	1.68 ± 0.34	0.704
E/A ratio	1.08 ± 0.41	1.04 ± 0.37	1.24 ± 0.54	1.14 ± 0.49	0.445
E/e’ ratio	9.64 ± 3.47	9.53 ± 3.31	10.69 ± 4.49	10.25 ± 3.73	0.206
Mitral deceleration time, ms	188 ± 63	184 ± 62	174 ± 42	212 ± 82	0.218
RV end-diastolic area, mm^2^/m^2^	11 (9–13)	10 (9–12)	10 (9–14)	13 (10–16)	0.536
RV end-systolic area, mm^2^/m^2^	7 (5–8)	6 (5–8)	7 (5–9)	7 (5–10)	0.574
RV systolic pressure, mmHg	35 ± 10	32 ± 9	35 ± 8	36 ± 8	0.958
TAPSE, mm	20 ± 3	21 ± 3	20 ± 2	20 ± 4	0.260
Right ventricular shortening fraction, %	43 ± 7	44 ± 6	42 ± 8	43 ± 9	0.707
Moderate mitral regurgitation, *n* (%)	32 (16)	18 (16)	7 (16)	7 (15)	0.956
Moderate tricuspid regurgitation, *n* (%)	5 (2.5)	3 (2.7)	1 (2.2)	1 (2.1)	0.841

Values are mean ± SD, median (IQR), or *n* (%). *P* value by Kruskal–Wallis or one-way ANOVA for non-Gaussian and Gaussian distributed continuous variables, respectively. *P* value by *χ*^2^ test for categorical variables (Bonferroni correction: *p < 0.05 vs seronegative; **p < 0.001 vs seronegative). Bold values indicate significant (<0.05) *p*-values.

Abbreviations: ACE, angiotensin converting enzyme; ARB, angiotensin II receptor blocker; BNP, brain natriuretic peptide; CRP, C-reactive protein; DBP, diastolic blood pressure; E/A, early/late filling velocity on transmitral Doppler; E/e'ratio, early filling velocity on transmitral Doppler/early relaxation velocity on tissue Doppler; eGFR, estimated glomerular filtration rate; HR, heart rate; LV, left ventricular; LVEDVi, left ventricular end diastolic volume index; LVEF, left ventricular ejection fraction; LVESVi, left ventricular end systolic volume index; PPCI, primary percutaneous coronary intervention; RV, right ventricle; SBP, systolic blood pressure; TAPSE, tricuspid annular plane systolic excursion.

### 3.2 Prognostic impact of AT1R-AAs and ETAR-AAs

During a median follow-up of 429 days (IQR 235–730 days), MACE occurred in 39 (19.5%) patients (22 cardiovascular-related deaths, 2 nonfatal myocardial re-infarctions, and 15 hospitalizations for heart failure).

**Figure 1:**
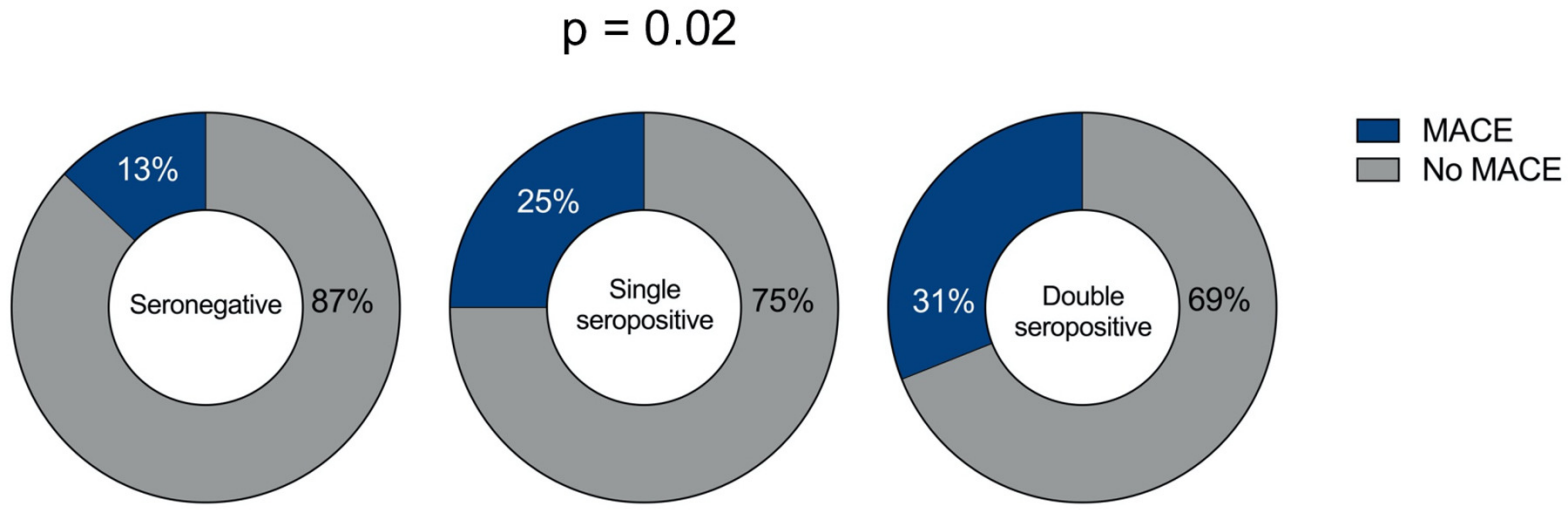
Frequency of MACE in STEMI patients based on serum autoantibody status. MACE in seronegative STEMI patients (left pie chart); MACE in single seropositive (AT1R-AAs or ETAR-AAs seropositive) STEMI patients (middle pie chart); and MACE in double seropositive (AT1R-AAs and ETAR-AAs seropositive) STEMI patients (right pie chart). The *P*-value for the difference between the groups is reported.

The incidence of MACE was 13% in seronegative patients, 25% in single-seropositive patients, and 31% in double-seropositive patients (*p* = 0.021) (Figure 1). The incidence of the outcome was higher in patients with double seropositivity than in seronegative patients (31% vs. 13%, OR 3.0, 95% CI 1.29–6.96, *p* = 0.009) and was also higher also in patients with single seropositivity than in seronegative ones (25% vs. 13%, OR 2.28, 95% CI 0.94–5.52, *p* = 0.06). The characteristics of the patients according to the occurrence of the endpoint are shown in Supplementary Table S1.

To further clarify the association between serum levels of AT1R-AAs/ETAR-AAs and the risk of MACE, multivariable logistic regression analysis was performed. After adjusting for confounding factors, the risk of MACE was related to both AT1R-AAs (OR 1.07, 95% CI 1.02–1.16, *p* = 0.03) and ETAR-AAs (OR 1.08, 95% CI 1.01–1.15, *p* = 0.01) serum levels (Supplementary Table S2).

**Figure 2:**
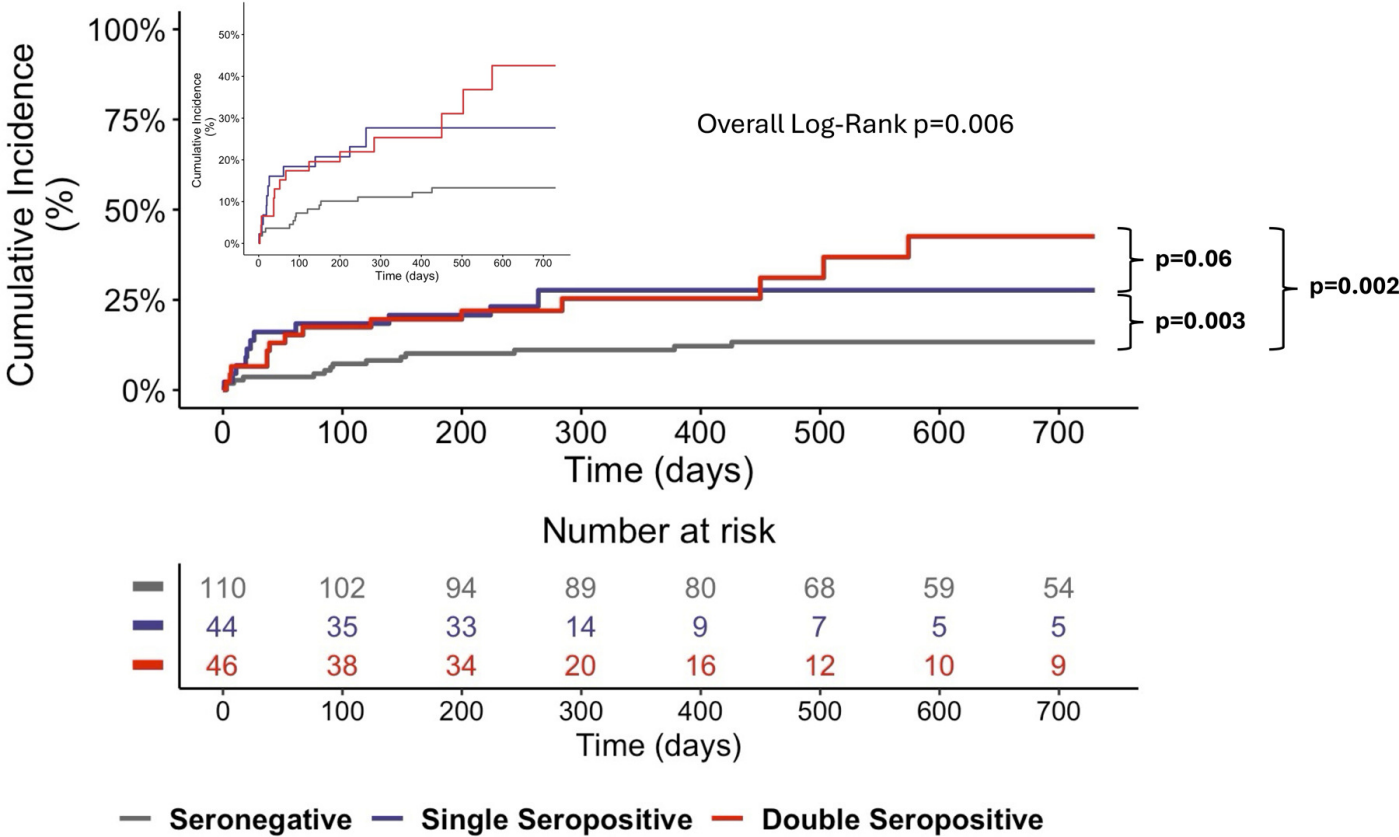
Kaplan–meier curves for MACE at the 4-year follow-up. The Kaplan–Meier curves show significantly higher MACE rates for single- and double-seropositive patients than for seronegative patients. The inset graph shows the data on an expanded *y* axis.

Survival analysis using Kaplan–Meier curves showed a significantly higher incidence of MACE in patients with single or double autoantibody seropositivity than in seronegative patients (Figure 2).

Univariable Cox regression analysis showed that autoantibody seropositivity (*p* = 0.009) was associated with an increased risk of MACE (Table 2). At multivariable Cox regression analysis, double seropositivity was independently associated with an increased risk of MACE (HR 2.386, 95% CI 1.471–3.864, *p* < 0.001) (Table 2). The *c-statistic* for the Cox multivariable model was 0.907 for MACE (SE 0.034, 95% CI 0.840–0.973; *p* < 0.0001), indicating a fair to good discriminatory ability.

**Table 2:** Univariable and multivariable Cox proportional hazard models for MACE (*n* = 200).

Variable	Univariable analysis		Multivariable analysis	
	Hazard ratio (95% CI)	*P* value	Hazard ratio (95% CI)	*P* value
Autoantibodies seropositivity		**0.009**		**0.002**
Single seropositivity vs. seronegative	2.646 (1.188–5.894)	**0**.**017**	1.573 (0.658–2.769)	0.748
Double seropositivity vs. seronegative	2.940 (1.394–6.200)	**0**.**005**	2.386 (1.471–3.864)	**<0**.**001**
Demographic characteristics				
Age, years	1.048 (1.015–1.088)	**0**.**004**	1.044 (0.954–1.142)	0.354
Male sex, *n* (%)	1.145 (0.441–2.975)	0.781		
Body surface area, m^2^	0.517 (0.320–0.865)	0.567		
Body mass index, kg/m^2^	1.029 (0.948–1.117)	0.490		
Medical history				
Obesity, *n* (%)	1.490 (0.630–3.524)	0.364		
Hypertension, *n* (%)	2.380 (1.222–4.633)	**0**.**011**	4.253 (0.835–5.649)	0.081
Diabetes mellitus, *n* (%)	1.347 (0.564–3.217)	0.503		
Hypercholesterolemia, *n* (%)	1.442 (0.669–3.107)	0.351		
Smoker, *n* (%)	1.976 (1.198–4.672)	0.121		
Ex-smoker, *n* (%)	0.961 (0.451–2.044)	0.917		
Coronary artery disease, *n* (%)	2.211 (0.837–5.839)	0.109		
Pacemaker, *n* (%)	1.002 (1.001–1.005)	0.638		
Chronic obstructive pulmonary disease, *n* (%)	4.679 (1.106–9.792)	**0**.**036**	1.081 (1.027–3.151)	**0**.**048**
Peripheral vascular disease, *n* (%)	1.852 (0.640–5.358)	0.255		
SBP at admission, mmHg	0.989 (0.973–1.005)	0.164		
DBP at admission, mmHg	0.989 (0.964–1.014)	0.368		
HR at admission, beats/min	1.017 (0.994–1.041)	0.153		
O_2_ sat at admission, %	0.922 (0.855–0.994)	**0**.**035**	1.042 (0.858–1.266)	0.679
Pain to balloon time, min	1.002 (1.001–1.004)	0.943		
Door to balloon time, min	1.001 (0.996–1.002)	0.963		
TIMI flow after PPCI <3	3.331 (1.342–8.267)	**0**.**009**	1.425 (0.569–3.492)	0.425
Blush grade after PPCI ≤2	4.758 (1.893–11.96)	**<0**.**001**	3.258 (1.732–6.917)	**0**.**007**
Laboratory values at admission				
Peak of troponin, ng/L	1.003 (1.001–1.006)	**<0**.**001**	1.015 (1.011–1.032)	**0**.**004**
CRP, mg/L	1.012 (1.005–1.018)	**<0**.**001**	1.013 (0.993–1.034)	0.212
BNP, pg/ml	1.002 (0.976–1.003)	0.213		
eGFR, ml/min	0.968 (0.952–0.984)	**<0**.**001**	0.978 (0.937–1.021)	0.309
D-dimer, ng/ml	1.004 (1.001–1.019)	**<0**.**001**	1.001 (1.000–1.002)	0.620
Hemoglobin, mmol/L	0.863 (0.778–0.888)	0.061		
Creatinine, μmol/L	1.004 (1.003–1.005)	**<0**.**001**		
Urea, mmol/L	1.011 (1.010–1.015)	**0**.**012**		
Medication				
ACE-inhibitor/ARB, *n* (%)	1.546 (0.677–3.533)	0.301		
β-blocker, *n* (%)	1.498 (0.518–4.332)	0.456		
Statin, *n* (%)	0.653 (0.155–2.757)	0.562		
Diuretic, *n* (%)	0.825 (0.195–3.484)	0.794		
Echocardiographic characteristics at discharge				
LVEDVi, ml/m^2^	1.023 (1.007–1.039)	**0**.**004**		
LVESVi, ml/m^2^	1.040 (1.020–1-061)	**<0**.**001**		
LVEF, %	0.932 (0.897–0.969)	**<0**.**001**	1.237 (1.028–1.489)	**0**.**024**
LVEF preserved ≥50%, *n* (%)	0.378 (0.159–0.899)	**0**.**028**		
LV Mass index, g/m^2^	1.020 (1.004–1.035)	**0**.**013**	1.012 (0.986–1.039)	0.359
Wall motion score index	9.206 (2.895–11.27)	**<0**.**001**	1.524 (1.052–1.890)	**0**.**006**
E/A ratio	0.979 (0.363–2.640)	0.967		
E/e’ ratio	1.095 (0.985–1.218)	0.094		
Mitral deceleration time, ms	0.999 (0.992–1.005)	0.702		
RV end-diastolic area, mm^2^/m^2^	0.953 (0.906–0.998)	0.158		
RV end-systolic area, mm^2^/m^2^	0.941 (0.901–0.991)	**0**.**041**		
RV systolic pressure, mmHg	1.002 (0.997–1.006)	0.259		
TAPSE at discharge, mm	0.712 (0.225–2.253)	0.563		
Right ventricular shortening fraction, %	0.951 (0.907–0.997)	**0**.**037**	0.875 (0.788–0.970)	**0**.**011**
Moderate mitral regurgitation, *n* (%)	1.003 (0.899–1.006)	0.236		
Moderate tricuspid regurgitation, *n* (%)	1.001 (0.966–1.003)	0.852		

Abbreviations: ACE, angiotensin converting enzyme; ARB, angiotensin II receptor blocker; BNP, brain natriuretic peptide; CRP, C-reactive protein; DBP, diastolic blood pressure; E/A, early/late filling velocity on transmitral Doppler; E/e'ratio, early filling velocity on transmitral Doppler/early relaxation velocity on tissue Doppler; eGFR, estimated glomerular filtration rate; HR, heart rate; LV, left ventricular; LVEDVi, left ventricular end diastolic volume index; LVEF, left ventricular ejection fraction; LVESVi, left ventricular end systolic volume index; PPCI, primary percutaneous coronary intervention; RV, right ventricle; SBP, systolic blood pressure; TAPSE, tricuspid annular plane systolic excursion.

**Figure 3:**
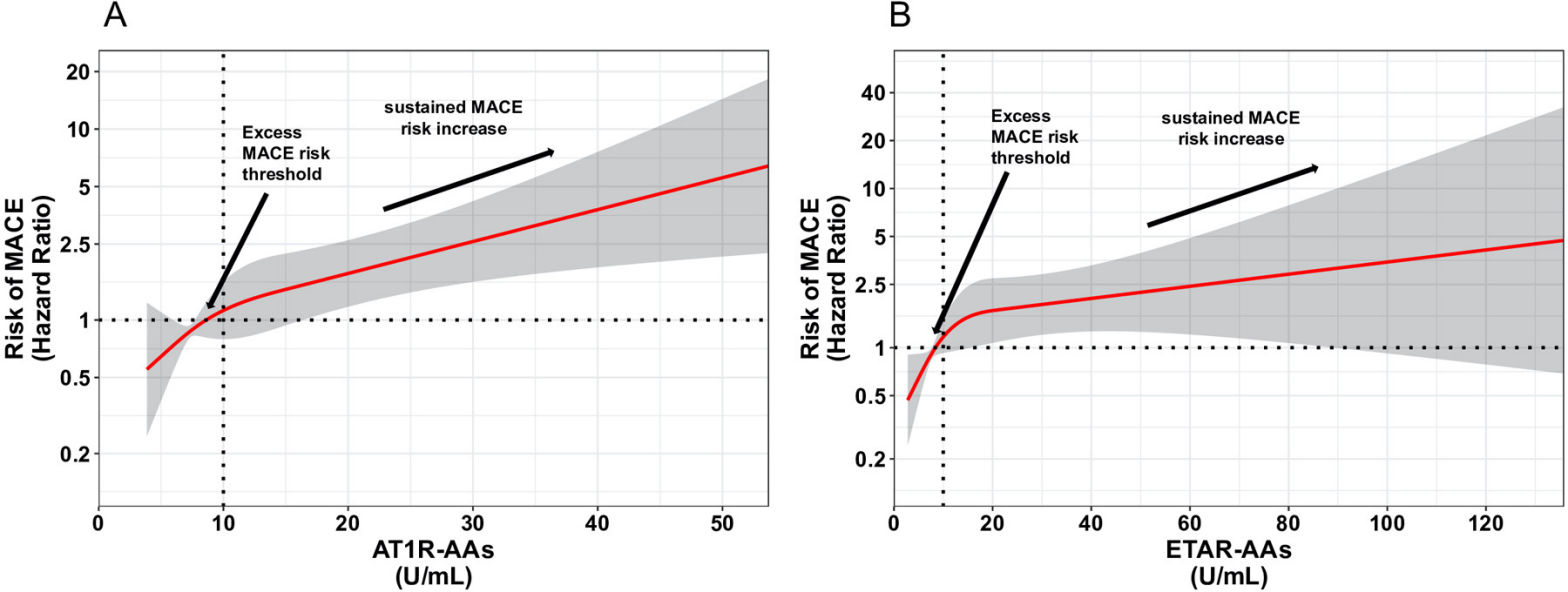
Spline curves for AT1R-AAs and ETAR-AAs versus MACE. Changes in the hazard ratio (HR) across the AT1R-AAs **(A)** and ETAR-AAs **(B)** are demonstrated in spline curves on a hazard scale with overlaid 95% confidence intervals (gray shaded area). This scale shows the relationship between AT1R-AAs, ETAR-AAs, and MACE.

Levels of AT1R-AAs and ETAR-AAs, when analyzed as continuous variables, were shown to correlate with a higher risk of experiencing MACE (Figure 3).

## 4 Discussion

In this study, we report, for the first time, the prognostic role of AT1R-AAs and ETAR-AAs in patients with STEMI. Ninety (45%) patients were seropositive for at least one autoantibody. Specifically, 44 (22%) patients were seropositive for AT1R-AAs or ETAR-AAs, and 46 (23%) patients were seropositive for both AT1R-AAs and ETAR-AAs. The incidence of MACE in AT1R-AAs- and ETAR-AA-seropositive patients was significantly higher than that in seronegative patients. During follow-up, the MACE risk was significantly higher in both single- and double-seropositive patients than in seronegative patients.

STEMI continues to be a global health issue, despite notable progress in its diagnosis and treatment. Furthermore, patients with STEMI undergoing PPCI have shown no improvement in myocardial infarction severity over the last few decades (3). Post-STEMI complications, including heart failure and recurrent infarction, can substantially affect patient prognosis. Timely recognition and treatment of these complications are essential for improving patient outcomes. In addition to comorbidities and myocardial infarction extension, the role of inflammation and fibrosis in STEMI outcomes has also received increasing attention (34, 35).

AT1R and ETAR are GPCRs expressed in different cell types, including vascular myocytes, immune cells, endothelial cells, and fibroblasts (36). Notably, AT1R and ETAR can be activated not only by their natural ligands but also by specific autoantibodies (AT1R-AAs and ETAR-AAs, respectively), which can bind AT1R and ETAR and regulate their function. AT1R-AAs and ETAR-AAs, which mirror (and amplify) the effects of the natural ligands AngII and ET1, induce vasoconstriction and stimulate pro-inflammatory and pro-fibrotic pathways (37, 38). Compared with AngII and ET1, receptor desensitization secondary to binding of AT1R-AAs and ETAR-AAs to their target receptors is more difficult, so that the autoantibodies can impose a sustained pathological stimulatory effect on receptors (8, 9, 11). Furthermore, the interaction between autoantibodies and receptors, combined with increased sensitivity to natural binding molecules, may contribute to an additional aspect of irregular vascular function (39). The concurrent presence of AT1R-AAs and ETAR-AAs also suggests the significance of receptor pairing, which could modify the impact of agonists on signal transmission (40).

The role of AT1R-AAs and ETAR-AAs in cardiovascular diseases is well established (9); however, their specific impact on outcomes after myocardial infarction has not been previously investigated. Two distinct processes, potentially initiated by AT1R-AAs and ETAR-AAs, and working together to promote myocardial fibrosis (41), play crucial roles in determining poorer outcomes following myocardial infarction: microvascular dysfunction (14) and myocardial inflammation (34). In terms of the inflammatory response, AT1R-AAs and ETAR-AAs are recognized for their ability to activate specific intracellular inflammatory cascades (8), which may contribute to the inflammatory process within the myocardium. Like AngII and ET1, AT1R-AAs and ETAR-AAs can indeed activate signal transduction pathways in non-immune and immune cells (8, 11, 42). Although the signaling molecules activated by AT1R-AAs and ETAR-AAs in non-immune cells are well characterized (42), the signaling pathways modulated by these autoantibodies in immune cells remain to be elucidated. Regarding microvascular dysfunction, the potential association with AT1R-AAs and ETAR-AAs is even more intriguing. Although the overall vasoconstrictive effects of these autoantibodies have long been recognized (8, 38), their particular impact on heart disease and coronary microcirculation has only recently become a focus of research (9, 12, 13). Notably, ETAR-AAs have been linked to the no-reflow phenomenon following acute myocardial infarction, a condition in which the coronary microcirculation becomes completely dysfunctional, hindering myocardial reperfusion (12). We have also recently demonstrated the association of AT1R-AAs and ETAR-AAs with post-infarction left ventricular remodeling (13). After myocardial infarction, both no-reflow and adverse left ventricular remodeling negatively influence clinical outcomes (14, 15). The association between AT1R-AAs and ETAR-AAs and these important determinants of myocardial infarction outcomes suggests that these autoantibodies may be involved in the prognosis of myocardial infarction and may explain the results of the present study. However, further investigation using preclinical and animal studies is required to confirm these hypotheses.

In this study, we used a unique approach to assess not only the levels of each autoantibody but also the overall degree of seropositivity. Thus, we did not only examine the effect of a single autoantibody but rather investigated the overall burden of anti-AT1R/ETAR autoimmunity, hypothesizing that the simultaneous presence of both autoantibodies exerts a larger effect by activating similar complementary intracellular pathways, which amplify their effects in a sort of “double hit” phenomenon. We decided to take this approach given (1) a high similarity of effects between the two autoantibodies (8, 38); (2) autoantibody-mediated receptor cross-talk (32, 43, 44); and (3) previous literature on the topic. For example, in preeclampsia, all patients have high levels of AT1R-AAs, but only those with severe disease are seropositive for ETAR-AAs (45). In pediatric kidney disease, the presence of both antibodies is significantly associated with arteritis, elevated inflammatory markers, and a decline in renal function, suggesting possible interaction effects (46). Notably, a combined effect has been observed with other autoantibodies, such as those targeting heat-shock protein 60 (anti-Hsp60). These antibodies enhance the likelihood of arterial vascular events only when antiphospholipid antibodies are also present (47). Similarly, in idiopathic nephrotic syndrome, circulating podocyte autoantibodies exert their full effect only once antibodies to glomerular endothelial cells have damaged the endothelial cells (48). Based on these previous findings, we believe that AT1R-AAs and ETAR-AAs have a cumulative effect, and that the simultaneous presence of both autoantibodies is associated with a higher probability of recurrent cardiovascular adverse events due to the amplification of the effects elicited by each single autoantibody.

AT1R-AAs and ETAR-AAs are both IgG autoantibodies. The autoantibodies detected during the acute stage of myocardial infarction are therefore likely to be pre-existing rather than induced by myocardial ischemia. AT1R-AAs and ETAR-AAs are compelling components of our immune system (9, 37, 38, 49). Pivotal to the scope of understanding no-reflow and adverse remodeling after myocardial infarction, as we recently described (12, 13), autoantibodies anti-GPCRs have recently been described as powerful markers of chronic GPCR expression, reflecting chronic individual exposure to different environmental factors (“Exposome”). This antibody network (“Antibodiom”) is thus shaped by both environmental exposure and an individual's immune system (49). Although speculative, we hypothesize that each patient possesses unique levels of AT1R-AAs and ETAR-AAs, which serve as a signature of their “exposome” and influence their reaction to an acute ischemic event and their susceptibility to no-reflow and left ventricular remodeling, regardless of infarct size (12, 13). This hypothesis aligns with emerging evidence suggesting the immune system's role in homeostasis beyond host defence (50). Additionally, specific metabolic conditions, such as those induced by ischemia and reperfusion, may be necessary for the full activity of AT1R-AAs and ETAR-AAs (51).

Following myocardial infarction, the endothelium produces AngII and ET1, which are not merely passive observers but indicators of infarct size and potential enhancers of AT1R-AAs and ETAR-AAs effects (52). This situation is further complicated by factors beyond natural ligands and autoantibodies. Studies on rat models have shown that ischemia and reperfusion lead to an increase in AngII and ET1 binding sites on cardiac membranes (53). Consequently, both ligands (elevated AngII and ET1 production and higher AT1R-AAs and ETAR-AAs levels) and receptors (increased AT1R and ETAR expression) can affect infarct extension and no-reflow.

As regards the potential clinical implications of our findings, the first and most straightforward one is that of having a biomarker that allows stratification of the risk of adverse events after STEMI. Seropositivity for AT1R-AAs/ETAR-AAs (and most of all double seropositivity) allows the identification, even in the first phases after the acute event, of STEMI patients with a risk of poor prognosis who could benefit from closer monitoring and follow-up. However, we believe that the potential implications of AT1R-AAs/ETAR-AAs in ischemic heart disease go beyond those of simple risk biomarkers. Our findings open the first window on the interconnection between autoimmunity and acute coronary syndromes, potentially leading to major changes in the treatment of patients with acute myocardial infarction. Currently, no available therapy specifically inhibits AT1R-AAs and ETAR-AAs and/or regulates the expression of AT1R and ETAR. Nevertheless, laboratory studies have shown that angiotensin receptor blockers (ARBs) and ETAR antagonists can counteract the stimulatory effects of AT1R-AAs and ETAR-AAs (54). Clinical research has also demonstrated that combining plasmapheresis with ARBs reduces the risk of acute rejection in transplant patients (55), and recent findings indicate that ARBs are linked to lower overall mortality and fewer heart failure hospitalizations than ACE inhibitors in myocardial infarction patients (56). Notably, a recent clinical trial also showed that sparsentan, a non-immunosuppressive, dual endothelin and angiotensin receptor antagonist with high selectivity for ETAR and AT1,R is highly effective in IgA nephropathy (57). This evidence supports the hypothesis that blocking the effects of AT1R-AAs/ETAR-AAs with already approved and well-tolerated drugs might be beneficial in patients with STEMI. Due to the observational design and limited sample size, the therapeutic implications remain speculative and warrant further investigation. However, advancing our understanding of the cell signaling pathways and mechanisms underlying autoantibody-induced pathology triggered by AT1R-AAs and ETAR-AAs could lead to the development of novel therapies. These treatments can specifically target autoantibody effects while preserving the normal physiological functions of the RAAS and endothelin systems.

This study had some limitations, including a small sample size, single center, single ethnicity, and short follow-up time. Moreover, although this is a common issue in studies investigating acute coronary events (58, 59), most patients enrolled in the present study were men. Although the current literature does not report sex-based differences in the effects of AT1R-AAs and ETAR-AAs, the autoimmune profile is significantly different between men and women (60); thus, our findings cannot be confidently extended to female patients. The relatively small number of events did not allow for the investigation of the association between autoantibody levels and the individual components of the primary outcome. Additionally, the observational nature of the study implies that residual confounding might be present; although we adjusted our multivariate survival analysis for variables associated with worse outcomes, unmeasured comorbidities/factors might still be present. Dynamic changes in antibody titers were not investigated, and we do not know whether the levels of autoantibodies changed during follow-up. We also investigated only medical therapy at discharge and did not know which medical therapy patients were prescribed during follow-up. However, given that all patients were followed up at the same center, we can hypothesize a homogeneous approach. Additionally, although the cutoff of 10 U/ml to define seropositivity has been widely used in previous studies, its accuracy in patients with cardiovascular disease should be tested in larger cohorts. Finally, the observational study design and correlations observed in this study do not allow for the inference of causality and should therefore be considered hypothesis-generating. To validate our hypothesis, ELISA tests for antibody detection are not sufficient. Additional preclinical studies are necessary to gain a deeper understanding of the molecular pathways through which AT1R-AAs and ETAR-AAs exert their effects. Future multicenter clinical studies with longer follow-up periods and larger cohorts of patients are warranted to validate our findings in independent patient cohorts and to capture events occurring beyond the early years following STEMI.

In conclusion, our research provides the first evidence suggesting that functional autoantibody-mediated vascular receptor activation plays a role in revascularized STEMI. Additionally, it suggests that AT1R-AAs and ETAR-AAs may serve as potential prognostic markers for acute myocardial infarction. These findings may lead to the development of innovative treatments to improve outcomes for STEMI patients.

## Data Availability

The datasets presented in this article are available from the corresponding author on reasonable request. Requests to access the datasets should be directed to Francesco Tona, francesco.tona@unipd.it.
