# Supplementary material for: Prognostic role of angiotensin-II receptor type 1 and endothelin-1 receptor type A agonistic autoantibodies in patients with acute myocardial infarction
Source: Front Cardiovasc Med. 2025 Aug 12;12:1515693. doi: 10.3389/fcvm.2025.1515693 (PMC12378766; doi:10.3389/fcvm.2025.1515693)
Supplement: Supplementary file 1 [file Datasheet1.docx]

Supplementary Material

# Supplemental Methods

## Echocardiography

TTE images were obtained with the patients at rest in the left lateral decubitus position using a commercially available ultrasound system (Vivid E9 and E95, GE Vingmed Ultrasound, Horten, Norway) equipped with M5S transducers in the parasternal and apical views, as previously described (1). Standard M-mode and 2D, colour, pulsed wave and continuous wave Doppler images were acquired and saved in cineloop format. Echocardiographic data were analyzed offline using the EchoPAC version 202 software (GE Vingmed Ultrasound, Horten, Norway).

## Laboratory Assays

After being drawn, plasma and serum samples were stored at -80 °C in appropriate tubes until further analysis. Serum samples (diluted 1:100), standards, and positive and negative controls were added to the plate and incubated at 4 °C for 2 h. After three washing steps, plates were incubated for 60 min at room temperature with horseradish peroxidase-labelled goat anti-human IgG, followed by incubation with 100 μL of the chromogenic substrate tetramethylbenzidine (TMB) for 20 min. After blocking the reaction, the absorbance of each well was measured at 450 nm using an ELISA reader (iEMS Reader MF Multiskan, Thermo). Each specimen was analyzed in duplicate. Standard curves with AT1R-AAs and ETAR-AAs standard points (2.5, 5, 10, 20, and 40 U/mL) were included in each plate to enable accurate quantification of antibodies in patient serum.

### Cut-off for seropositivity

As the role of AT1R-AAs and ETAR-AAs has never been assessed in the context of acute myocardial infarction, there are no cutoffs specifically tailored for this condition. In accordance with our immunologists, we used the cutoffs recommended by the manufacturer of the ELISA kit for the determination of AAs levels (CellTrend, Luckenwalde, Germany), which suggest that levels ≤10 U/mL should be considered as negative, levels >10 and ≤17 should be considered borderline/mild positive and levels >17 should be considered frankly positive. Given the exploratory nature of our study, we decided to adopt the threshold of 10 U/mL to define seropositivity. In future studies with more patients, we will also be able to test the threshold of 17 U/mL and assess whether it increases the accuracy of our model.

As reported in the Methods, the choice of 10 U/mL as cutoff is also supported by previous literature. The largest study analyzing the role of AT1R-AAs and ETAR-AAs in cardiovascular disease (2) has not specifically reported the thresholds used to define seropositivity but stated that they were defined according to kit manufacturer recommendations (CellTrend, as we did). Other studies used ROC-derived cutoffs. These cutoffs were derived from each study population and not validated in external cohorts. Interestingly, a large study on systemic sclerosis found 9.2 and 10.4 U/mL as best cutoffs for AT1R-AAs and ETAR-AAs, very close to 10 U/mL suggested by the manufacturer (3,4).

Other studies used similar cut-offs, but the manufacturer of the kit is different and thus the results might be non-comparable.

## Statistical Analysis

Given the lack of previous studies on this topic, we could not a priori calculate the sample size required to test our hypotheses. Indeed, while we previously investigated the odds of MACE associated with AT1R-AAs and ETAR-AAs seropositivity, there are no currently published data about 1) the prevalence of none, single, or double AAs seropositivity; and 2) the risk of MACE associated with each of these groups in a time to event model.

The post-hoc power analysis (calculated using the R package “powerSurvEpi”) showed that the time to event analysis of double seropositive versus seronegative patients achieved a statistical power of 85.2% with a significance level (alpha) of 0.05. For the time to event analysis of single seropositive versus seronegative patients, the achieved power was 72.0% with an alpha of 0.05.

1. Tona F, Civieri G, Vadori M, Masiero G, Iop L, Marra MP, Perin V, Cuciz E, Cecere A, Bernava G, et al. Association of Angiotensin II Receptor Type 1 and Endothelin-1 Receptor Type A Agonistic Autoantibodies With Adverse Remodeling and Cardiovascular Events After Acute Myocardial Infarction. *J Am Heart Assoc* (2024)e032672. doi: 10.1161/JAHA.123.032672

2. Hall J, Bourne KM, Vernino S, Hamrefors V, Kharraziha I, Nilsson J, Sheldon RS, Fedorowski A, Raj SR. Detection of G Protein–Coupled Receptor Autoantibodies in Postural Orthostatic Tachycardia Syndrome Using Standard Methodology. *Circulation* (2022) 146:613–622. doi: 10.1161/CIRCULATIONAHA.122.059971

3. Riemekasten G, Philippe A, Näther M, Slowinski T, Müller DN, Heidecke H, Matucci-Cerinic M, Czirják L, Lukitsch I, Becker M, et al. Involvement of functional autoantibodies against vascular receptors in systemic sclerosis. *Ann Rheum Dis* (2011) 70:530–536. doi: 10.1136/ard.2010.135772

4. Becker MO, Kill A, Kutsche M, Guenther J, Rose A, Tabeling C, Witzenrath M, Kühl AA, Heidecke H, Ghofrani HA, et al. Vascular Receptor Autoantibodies in Pulmonary Arterial Hypertension Associated with Systemic Sclerosis. *Am J Respir Crit Care Med* (2014) 190:808–817. doi: 10.1164/rccm.201403-0442OC

# Supplemental Tables

## Supplemental Table 1: Clinical and Laboratory Data for Patients With and Without MACE (n=200)

|  | **No MACE**  **(n=161)** | **MACE**  **(n=39)** | **p** |
| --- | --- | --- | --- |
| Demographic characteristics | | | |
| Age, years | 61 ± 11 | 67 ± 9 | **0.001** |
| Male sex, n (%) | 136 (84) | 30 (77) | 0.260 |
| Body surface area, m^2^ | 1.8 ± 0.1 | 1.9 ± 0.2 | 0.625 |
| Body mass index, kg/m^2^ | 26 ± 4 | 27 ± 5 | 0.523 |
| Medical history | | | |
| Obesity, n (%) | 22 (14) | 7 (18) | 0.328 |
| Hypertension, n (%) | 71(44) | 26 (66) | **0.011** |
| Diabetes mellitus, n (%) | 17 (10) | 6 (15) | 0.397 |
| Hypercholesterolemia, n (%) | 39 (24) | 11 (28) | 0.353 |
| Smoker, n (%) | 52 (32) | 7 (18) | **0.031** |
| Ex-smoker, n (%) | 65 (40) | 14 (36) | 0.957 |
| Coronary artery disease, n (%) | 10 (6) | 5 (12) | 0.082 |
| Pacemaker, n (%) | 4 (2) | 1 (2) | 0.953 |
| Chronic obstructive pulmonary disease, n (%) | 1 (0.6) | 2 (5) | **0.026** |
| Peripheral vascular disease, n (%) | 9 (6) | 4 (10) | 0.205 |
| SBP at admission, mmHg | 137 ± 23 | 131 ± 28 | 0.271 |
| DBP at admission, mmHg | 81 ± 14 | 79 ± 20 | 0.448 |
| HR at admission, beats/min | 80 ± 16 | 85 ± 14 | 0.121 |
| O_2_ saturation at admission, % | 98 ± 2 | 96 ± 4 | **0.034** |
| Pain to balloon time, min | 200 (125-391) | 197(113-403) | 0.940 |
| Door to balloon time, min | 75 (47-125) | 87 (48-166) | 0.963 |
| TIMI flow after PPCI < 3 | 13 (8) | 7 (18) | **0.015** |
| Blush grade after PPCI ≤ 2 | 9 (5) | 7 (18) | **0.001** |
| Laboratory values at admission | | | |
| Peak of troponin, ng/L | 59,262 (23,848-108,725) | 125,500 (57,490-278,800) | **0.002** |
| CRP, mg/L | 5.3 (3.0-13.3) | 11.7 (3.0-29.2) | 0.286 |
| BNP, pg/mL | 111 (47-213) | 226 (94-421) | 0.131 |
| eGFR, mL/min | 93 (83-101) | 89 (66-98) | **0.047** |
| D-dimer, ng/mL | 150 (150-201) | 298 (150-1,834) | **<0.001** |
| Hemoglobin, mmol/L | 7.9 (6.9-8.8) | 8.8 (7.1-9.1) | 0.312 |
| Creatinine, μmol/L | 93 (80-115) | 96 (81-117) | 0.512 |
| Urea, mmol/L | 7.2 (6.4-10.3) | 7.9 (6.2-10.9) | 0.369 |
| Medication at discharge | | | |
| ACE-inhibitor/ARB, n (%) | 143 (89) | 39 (100) | 0.329 |
| β-blocker, n (%) | 101 (63) | 33 (84) | 0.215 |
| Statin, n (%) | 161 (100) | 39 (100) | 1.000 |
| Diuretic, n (%) | 10 (6) | 2 (5) | 0.909 |
| Echocardiographic characteristics at discharge | | | |
| LVEDVi, mL/m^2^ | 56 ± 10 | 65 ± 22 | **0.003** |
| LVESVi, mL/m^2^ | 30 ± 9 | 38 ± 17 | **<0.001** |
| LVEF, % | 49 ± 9 | 41 ± 11 | **0.005** |
| LVEF preserved ≥ 50%, n (%) | 64 (40) | 7 (18) | **0.022** |
| LV Mass index, g/m^2^ | 95± 23 | 108 ± 26 | **0.034** |
| Wall motion score index | 1.64 ± 0.32 | 1.93 ± 0.43 | **0.005** |
| E/A ratio | 1.08 ± 0.39 | 1.08 ± 0.48 | 0.968 |
| E/e’ ratio | 9.4 ± 3.3 | 10.4 ± 4 | 0.127 |
| Mitral deceleration time, ms | 189 ± 66 | 184 ± 51 | 0.675 |
| RV end-diastolic area, mm^2^/m^2^ | 11 (8-13) | 12 (9-13) | 0.237 |
| RV end-systolic area, mm^2^/m^2^ | 8 (6-11) | 10 (7-12) | **0.033** |
| RV systolic pressure, mmHg | 30 ± 8 | 35 ± 10 | 0.624 |
| TAPSE, mm | 2.07 ± 0.35 | 2.02 ± 0.46 | 0.586 |
| Right ventricular shortening fraction, n (%) | 44 ± 7 | 40 ± 9 | **0.035** |
| Moderate mitral regurgitation, n (%) | 27 (17) | 5 (13) | 0.623 |
| Moderate tricuspid regurgitation, n (%) | 4 (2) | 1 (2) | 0.782 |

Abbreviations as in Table 1. Values are mean ± SD, median (IQR), or n (%).

## Supplemental Table 2: Logistic Regression Analysis of AT1R-AAs, and ETAR-AAs Serum Level and Outcome in Patients With STEMI (n = 200)

|  | **β Coefficient (SE)** | **OR (95% CI)** | **p value** |
| --- | --- | --- | --- |
| **AT1R-AAs** |  |  |  |
| Unadjusted | 0.060 (0.018) | 1.062 (1.025-1.101) | **0.001** |
| Adjusted^a^ | 0.073 (0.039) | 1.076 (1.021-1.162) | **0.031** |
| **ETAR-AAs** |  |  |  |
| Unadjusted | 0.018 (0.009) | 1.018 (1.012-1.036) | **0.036** |
| Adjusted^a^ | 0.077 (0.032) | 1.080 (1.014-1.150) | **0.016** |

^a^Adjusted for baseline characteristics that showed a univariate relationship with MACE (p < 0.05) after checking for collinearity: age, peak of troponin ng/L, hypertension (%), left ventricular ejection fraction at discharge (%), wall motion score index, blush grade ≤ 2 (%) and eGFR, mL/min at admission.
